# Natural pH Gradients in Hydrothermal Alkali Vents Were Unlikely to Have Played a Role in the Origin of Life

**DOI:** 10.1007/s00239-016-9756-6

**Published:** 2016-08-17

**Authors:** J. Baz Jackson

**Affiliations:** School of Biosciences, University of Birmingham, Birmingham, B15 2TT UK

**Keywords:** Natural pH gradient, Alkali vents, Chemiosmotic theory, Origin of life

## Abstract

The hypothesis that a natural pH gradient across inorganic membranes lying between the ocean and fluid issuing from hydrothermal alkali vents provided energy to drive chemical reactions during the origin of life has an attractive parallel with chemiosmotic ATP synthesis in present-day organisms. However, arguments raised in this review suggest that such natural pH gradients are unlikely to have played a part in life’s origin. There is as yet no evidence for thin inorganic membranes holding sharp pH gradients in modern hydrothermal alkali vents at Lost City near the Mid-Atlantic Ridge. Proposed models of non-protein forms of the H^+^-pyrophosphate synthase that could have functioned as a molecular machine utilizing the energy of a natural pH gradient are unsatisfactory. Some hypothetical designs of non-protein motors utilizing a natural pH gradient to drive redox reactions are plausible but complex, and such motors are deemed unlikely to have assembled by chance in prebiotic times. Small molecular motors comprising a few hundred atoms would have been unable to function in the relatively thick (>1 μm) inorganic membranes that have hitherto been used as descriptive models for the natural pH gradient hypothesis. Alternative hypotheses for the evolution of chemiosmotic systems following the emergence of error-prone gene replication and translation are more likely to be correct.

## Introduction

It has been suggested that “natural pH gradients” across inorganic membranes provided the energy needed to drive chemical reactions at the start of life on earth. Variants of the idea were presented in a number of publications by three overlapping research groups between 1993 and the present (Russell et al. 1993, 2010, 2014; Russell and Hall 1997, 2002; Martin and Russell 2003, 2007; Russell 2007; Martin et al. 2008, 2014; Nitschke and Russell 2009; Lane et al. 2010; Lane and Martin 2012; Martin 2012; Sousa et al. 2013; Sojo et al. 2014, 2016), and these have been extensively cited but not critically reviewed in the literature. It is said by these research groups that modern-day, hydrothermal alkali vents at “Lost City” near the mid-Atlantic ridge (Kelley et al. 2001, 2002, 2005; Ludwig et al. 2006) provide a model for understanding the natural pH gradients that might have existed across inorganic membranes in prebiotic times. The idea of a natural pH gradient driving primeval chemical reactions is made attractive by the fact that chemiosmotic coupling, as first described by Peter Mitchell, provides the major pathway for ATP synthesis (and other energy-requiring processes) in extant living organisms (Mitchell 1966, 1968).

In this context, the principles of chemiosmotic theory are here briefly reviewed, and the possibility that natural pH gradients could have generated useful chemical work at the origin of life is examined.

## Chemiosmotic Coupling and Natural pH Gradients

**Fig. 1:**
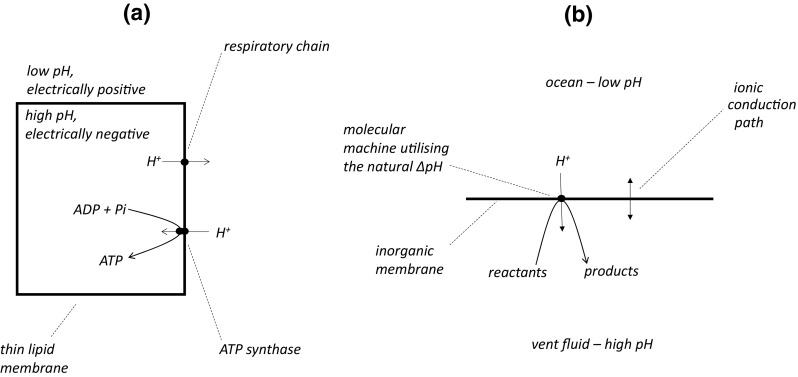
The principles of chemiosmotic coupling and of the natural pH gradient hypothesis. **a** The *rectangle* signifies either the inner membrane of a mitochondrion or the cytoplasmic membrane of a bacterium. **b** The *horizontal line* signifies an inorganic membrane separating the alkali vent fluid and the Hadean ocean. The *straight*, *single-headed arrows* in both parts indicate the direction of proton translocation. The *curved arrows* denote reactions catalysed by molecular machines. In the natural pH gradient hypothesis, (**b**) the reaction is shown as taking place in the vent but might alternatively proceed in the ocean. The *double-headed arrow* in (**b**) indicates the electrophoretic ion flux needed to relieve the unfavourable Δ*φ* resulting from charge transfer (either electrons or protons) through the molecular machine (see *text*)

Redox reactions taking place in respiratory and photosynthetic electron transport chains drive, or pump, hydrogen ions (protons, H^+^) across the membranes of mitochondria, chloroplasts and bacteria, and thus generate a proton electrochemical gradient (also called a protonmotive force, Δ*p*)—see Fig. 1a. The Δ*p* has two components, a pH gradient (ΔpH) arising from the resulting difference in H^+^ concentration between the aqueous phases on either side of the membrane, and an electrical potential gradient (or membrane potential, Δ*φ*) arising from the movement of charge across the membrane (e.g. the charge on the proton). Following Mitchell (1966, 1968):

1 $$\Delta p = \Delta \varphi - 0.062 \times \Delta {\text{pH}},$$

where Δ*p*, Δ*φ* and 0.062 × ΔpH are all expressed in volts. The conversion of ΔpH is achieved since 2.303 × RT/F is approximately 0.062 V at 40 °C. See Nicholls and Ferguson (2013) for explanatory detail. A number of factors can affect the relative contributions of Δ*φ* and ΔpH to Δ*p* across modern membranes. For example, in animal mitochondria, Δ*φ* often makes a greater contribution than ΔpH, but in the thylakoid membranes of chloroplasts, it is the smaller component.

In the subsequent steps, H^+^ is driven by Δ*p* back across the membrane, and the free energy of the gradient can thus be made available to membrane-embedded molecular machines, proteins such as the H^+^-ATP synthase which produces ATP from ADP and inorganic phosphate. The free energy available to the molecular machine from Δ*p* is given by

2 $$\Delta G \, = \, - \, F \, \Delta p,$$

where Δ*G* is in J mol^−1^ protons transported.

Thus, Δ*p* is generated by respiratory and photosynthetic electron transport chains, and consumed by the H^+^-ATP synthase. A chemiosmotic proton circuit across the membrane conserves the energy released by the electron transport chains in ATP synthesis—Fig. 1a.

In the hypothesis of Russell, Martin, Lane (RML) and colleagues, there is no active proton pumping. The two sides of an inorganic membrane are bathed by solutions of different pH—see Fig. 1b. On one side is an alkaline fluid (pH 9–11) issuing from an ocean floor vent; the alkalinity may be the result of serpentinization, the conversion of peridotite to serpentinite in the earth’s crust (Kelley et al. 2001). On the other side of the membrane is the ocean water that is thought to have been slightly acidic (pH6) in the Hadean era due to high concentrations of CO_2_ in the earth’s atmosphere (Russell et al. 1993). This gives rise to the natural pH gradient. Importantly, there is no electric potential difference across the membrane in this arrangement (Δ*φ* = 0), since charge has not been moved between the two aqueous phases. Thus, from Eq. (1), Δ*p* has only a ΔpH component (and Δ*p* = −0.062 × ΔpH). In some publications (Lane et al. 2010; Lane and Martin 2012; Sousa et al. 2013), the relatively acidic aqueous phase on the ocean side of the membrane is described as having a positive charge, and the alkaline, vent phase, a negative charge. This is an error: there is no electric potential difference across the membranes described in these formulations.

The RML groups sometimes appear to adopt a non-standard definition of the proton electrochemical gradient (Russell and Hall 1997, 2002). Representing it in Russell and Hall (2002) as Δ_*p*_, (rather than the more conventional Δ*p* (Mitchell 1968), it is expressed (compare Eq. 1) as follows:

3 $$\Delta_{p} = \, \Delta \varphi \, + \, \Delta E_{\text{pH}},$$

where Δ*E*_pH_ is defined as the “potential due to ambient pH” without further explanation. The term is puzzling since comparison with Eq. (1) puts Δ*E*_pH_ equal to −(2.303 × *RT*/*F*) × ΔpH, and because Eh usually represents the redox potential of a couple relative to the standard hydrogen electrode. Indeed, the term Δ*E*_pH_ in Russell and Hall (2002) is shown on a Pourbaix diagram relative to an ordinate labelled Eh, the redox potential. Despite implications to the contrary (Russell and Hall 1997), it must be emphasized that the definitions of Δ*φ* and ΔpH are independent of the oxidation–reduction chemistry of H^+^/H_2_, and that of other redox couples (Mitchell 1966, 1968; Nicholls and Ferguson 2013).

Leaving these idiosyncrasies to one side, and reverting to conventional formulations, it may be accepted that a natural pH gradient can, in principle, drive H^+^ across an inorganic membrane from the ocean side to the vent side. An appropriately constructed molecular machine located in the membrane could thus have been driven by the natural pH gradient and performed chemical work that might have been of value in the origin of life (Fig. 1b). If the ΔpH were 4 units (Δ*p* = 0.25 V), then the maximum energy available to drive the molecular machine would have been 24 kJ mol^−1^ of protons transferred, which is a significant value in a biological and, presumably, a prebiotic context.

During its operation, H^+^ would have been (formally) translocated through the machine from the ocean to the vent. This flow of protons driven by ΔpH would have been coupled to a useful chemical reaction, but simultaneously, since it involves the movement of charge across the membrane, would have resulted in the build-up of an electical potential, positive on the vent side, that would have *opposed* further turnover of the machine. To dissipate this unfavourable Δ*φ* and allow further turnover, the inorganic membrane must have been permeable to other ions that were prevalent in the local environment, hence the ionic conduction path shown in Fig. 1b. Mitchell and colleagues recognized this principle in their experiments on the synthesis of ATP in mitochondria using an “artificial” ΔpH (Reid et al. 1966). Despite a statement to the contrary (Sojo et al. 2016), the build-up of the unfavourable Δ*φ* would not have been significantly dissipated by “mixing [of aqueous solutions] elsewhere in the system”, since the electric potential gradient is influenced only by charge movement across the membrane.

The RML groups have written at great length on their views of other parallel developments taking place at the time natural pH gradients were supposedly involved in the origin of life, and of the later evolution of bioenergetics processes and metabolism. These interesting subjects will not be pursued here. Lest there should be any blurring of ideas relevant perhaps to different periods of time, it is emphasized that the present review is concerned only with the natural pH gradient hypothesis as it has been applied to early events at the origin of life (Fig. 1b), and two basic features of the hypothesis are therefore stressed. Firstly, it should be made clear that if the inorganic membrane were completely impermeable to H^+^, the molecular machine would operate efficiently. There would be no increase in its efficiency if the background proton permeability of the membrane were raised—compare with Sojo et al. (2014). Secondly, there would be no advantage accorded to the natural ΔpH-driven molecular machine (Fig. 1b) of having a Na^+^/H^+^ antiporter in the same membrane—compare with Sousa et al. (2013).

## Was a Natural pH Gradient Essential to the Origin of Life?

It is forcefully argued by Lane et al. (2010) that “chemiosmosis was central to the origin of life”. These authors conclude it is “nearly impossible” to see how life could have begun in the absence of natural pH gradients, and that “chemiosmosis was necessary”. The justification for this uncompromising view seems partly to stem from the RML groups’ own definition of primordial soup. They imply, but do not explore in rigorous detail, that chemical reactions in the primordial soup would have reached thermodynamic equilibrium, and that further steps on the chemical pathways leading to living organisms would have become impossible without energetic input from a natural pH gradient. The idea is at its most extreme (and startling) in Martin and Russell (2003) where, of course by analogy, it is said that “once autoclaved a bowl of chicken soup at any temperature will never bring forth life”. The RML perspective is probably not at all justified on thermodynamic grounds since we do not know enough even approximately to define the prebiotic system. The question should not have been, “how a solution at equilibrium can start doing chemistry” (Martin and Russell 2003), but, as others have asked, what was the likely chemical composition of the prebiotic soup and the physical factors acting upon it? Moreover, the perspective of the RML groups stands in contradiction to a large body of work in which candidate prebiotic reactions are carried out in the laboratory. For example, the recent experiments from Sutherland’s group lead to a description of the synthesis of activated nucleotides and a range of amino acids with moderately high yields from plausible prebiotic precursors in a plausible prebiotic environment (Powner et al. 2009; Patel et al. 2015), and it is well established that activated nucleotides can polymerize on mineral surfaces (Ferris et al. 1996). Within the confines of the RNA world hypothesis (Gilbert 1986), there are still formidable difficulties in understanding how an RNA replicase might have arisen from a primordial soup (Joyce 2012) but, in principle, there are no thermodynamic obstacles. The argument will not be further pursued here, except to counter-assert that it cannot reasonably be claimed on thermodynamic grounds that a natural pH gradient was indispensable at the origin of life. A balanced view of the issues associated with energy transformations at the start of life is given by Deamer and Weber (2010).

## Membrane Structures Holding Natural pH Gradients at the Origin of Life

The difficulties of constructing an interposing, functionally chemiosmotic, inorganic membrane between an alkali vent and the ocean should not be underestimated. The membrane must have appropriate mechanical and osmotic properties, it must be stable, there must be a geochemical mechanism for synthesizing the membrane and a local supply of its constituents.

**Fig. 2:**
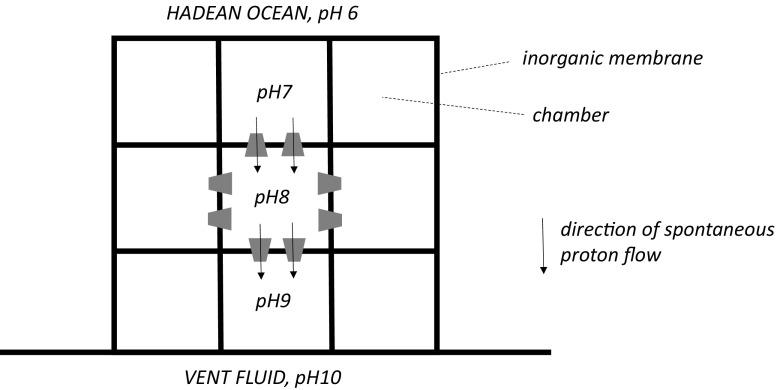
Reduced driving force and counterproductive proton flux in the multi-chambered version of the natural pH gradient hypothesis. Re-drawn from Martin and Russell (2003) and Sousa et al. (2013). Adjacent chambers share thick inorganic membranes. Illustrative values of internal pH are given for three chambers. The *trapezoids* represent molecular machines. They are shown only in the membrane of a single chamber, and they all face in the same direction with respect to that chamber. Spontaneous proton flux (from low to high pH) through the molecular machines is shown only in membranes of the representative chamber. The driving force, ΔpH, is +1 unit and −1 unit in vertically opposed membranes of the chamber

The RML research groups have put forward five different proposals for the way in which inorganic membranes might have been interposed between the vent fluids and the ocean. In early articles (Russell et al. 1993; Russell and Hall 1997), it was suggested that a “botryoidal” (grape like) system of interconnected chambers grew by hydrothermal inflation, and filled with alkaline vent fluid from the sea floor. The pH gradient was formed across the inorganic membrane surrounding the chambers. Later articles depict a system with many, mainly isolated but contacting chambers, with shared, inorganic membranes (Martin and Russell 2003; Sousa et al. 2013). In parenthesis it is noted that, although this model is not described explicitly, it may have two fundamental flaws (shown diagrammatically in Fig. 2). Firstly, the pH drop between the vent fluid and the ocean will be shared across multiple adjacent chambers, and this results in a reduced driving force for any one membrane-embedded, ΔpH-utilizing, molecular machine. Secondly, molecular machines with the same orientation in the membrane of a chamber but on opposite sides of that chamber will experience a ΔpH of opposite polarity—the two sets of machines will tend to be driven counterproductively in different chemical directions. In Russell (2007), Nitschke and Russell (2009) and Russell et al. (2010), a third picture is shown for an interposed inorganic membrane between an alkaline vent and the ocean—mounds of vertical conduits carry vent fluid into the ocean but some are plugged, and vectorial (e.g. transmembrane) chemistry linked to proton transport driven by ΔpH takes place in the plugs. In the fourth proposal, perhaps the simplest, an inorganic membrane lying on top of the alkaline fluid and underneath the ocean water blebs upwards and outwards to form a single compartment whose internal aqueous phase is continuous with that of the vent (Lane et al. 2010; Sojo et al. 2016). In Sojo et al. (2014) and Sojo et al. (2016), a fifth and rather a sophisticated model, suggested to be further along the path to protocells, has a *lipid* membrane enclosing a vesicle that is lodged and sealed into a hole in a planar, inorganic membrane that separates the vent fluid and the ocean: one half-shell of the vesicle membrane is therefore exposed to the ocean water and the other to the vent water. An equivalent criticism to that raised above is that those molecular machines located in the ocean half-shell of the vesicle membrane would experience a driving force of opposite polarity to those with similar orientation (with respect to the vesicle) in the vent half-shell, and the reactions would be counterproductively driven in different directions.

There is no evidence for any of these membrane systems at Lost City, no *mention* of any membrane-like structures (Kelley et al. 2001, 2002, 2005; Ludwig et al. 2006). Even the general assertion (Sojo et al. 2016) that alkali fluids from the vent and ocean water mix within a microporous labyrinth “producing steep gradients of … pH… across … inorganic barriers” does not, in fact, receive any support from the observations at Lost City.

### The Character of Membranes Suggested to Have Held a Natural pH Gradient at the Origin of Life

The molecular machines of modern-day organisms are located in *thin* membranes, lipid bilayers, approximately 5 nm across. The machines are proteins which *completely span* their membrane ensuring they make effective use of the full proton electrochemical gradient.

Consistently, however, the membrane described by the RML groups as separating the alkaline vent fluids from the acidic Hadean ocean, and housing the prebiotic molecular machines, was *thick* and inorganic (Russell and Hall 1997; Lane and Martin 2012). There is, in fact, good experimental evidence that long-chain fatty acids can be synthesized under plausible prebiotic conditions (McCollom et al. 1999), and that these molecules can spontaneously assemble to form thin, lipid bilayers surrounding vesicle structures (Mansy et al. 2008). The RML groups have however eschewed a role for such lipid bilayers in their natural pH gradient hypothesis. Rather, the thick and inorganic membrane may have suggested itself perhaps because of the probable vulnerability and fragility of lipid bilayers in the vent environment, especially as a consequence of hydrothermal and other convection currents, and because of the difficulties in assembling thin membrane structures of *large surface area* in the vents (see above).

Observations from submersibles by Kelley et al. (2005; Ludwig et al. 2006) indicate that the ocean floor mineral environment of the vents at Lost City is dominated by calcite and aragonite (both forms of calcium carbonate), and brucite (magnesium hydroxide) precipitated as the alkaline vent fluid mixes with the ocean. The precipitates are described as feathery, highly porous and extremely friable; they form delicate, finger-like masses and chimneys and flanges around the vent openings. It is difficult to see how these materials could serve as constituents of membranes separating vent from ocean, and be suitable for hosting chemiosmotic molecular machines driven by proton currents.

However, the RML groups argue that the ferrous iron content of the Hadean ocean was greater than it is today and, departing a little from the notion that Lost City is a good model for the natural pH gradient hypothesis, they opt for early-earth vent membranes with incorporated Fe(Ni)S and silicate (Russell et al. 1993; Russell and Hall 1997; Russell et al. 2010; Lane et al. 2010; Sojo et al. 2016)—note that silicate is only a trace component within the chimney materials at Lost City, and no sulphide was detected (Kelley et al. 2002). In their departure from the Lost City analogy, the RML groups suggest that the chemiosmotically-active, vent-ocean barriers at the onset of life may have resembled the gelatinous membranes generated in “chemical gardens” (Mee 1950), and the “bubbly membranes” produced, for example, when sodium sulphide is added to solutions of ferrous chloride (Russell et al. 1989; Russell and Hall 1997). Iron (and other metal) sulphide precipitates produced in the vent exhalations were said then to nucleate the membrane. The structures of the FeS membranes reported by the RML groups, and their geochemical context, have been questioned (Wächtershäuser 2006; Russell 2007). Moreover, a detailed experimental characterisation of the structure, and mechanical and osmotic properties of these candidate FeS membranes, put into the context of the natural pH gradient hypothesis, is still awaited more than 20 years after their suggestion was first mooted. Their value as a model for the hypothesis is still very much in doubt.

The thickness of these inorganic membranes is critical for a proper understanding of the way in which a natural pH gradient might drive a molecular machine. Electron micrographs of the membranes surrounding the iron monosulfide bubbles (Russell and Hall 1997; Martin and Russell 2007) indicate, to this reviewer, a thickness between 2 and 5 μm. Sojo et al. 2016 take the membrane thickness estimated in those earlier publications to be 1 μm, and we can accept this for present discussion. Crucially, these estimated thicknesses are >200 times greater than those of the lipid bilayers functioning in the chemiosmotic proton circuit of modern membranes. The continued, routine description of these inorganic membranes/barriers as “thin” by the RML groups is highly inappropriate.

Citing Kelley et al. (2001, 2005) and reverting from the FeS membrane view to the Lost City analogy, Lane and Martin (2012) describe “thin mineral walls” forming “osmotic barriers” between the alkali vents and the ocean. This seems to be a reinterpretation of the oceanography papers. Furthermore, purported thin mineral walls of structures at Lost City are described (Lane and Martin 2012) as being 100 nm–5 μm in “diameter”—presumably a typographical error for thickness—but it is difficult to find any support for this quoted range of barrier thickness in Kelley et al. (2001, 2005). Nevertheless, even a 100-nm barrier is some 20 times the thickness of a lipid bilayer. We shall return to this in the later section.

## ΔpH-Utilizing Molecular Machines

It is implicit throughout the work of the RML groups that the involvement of a natural ΔpH at the very origin of life rules out the possibility that the first molecular machines to use the energy of that gradient were proteins: in their view, gene encoded proteins (and ribozymes) are thought to have arrived much later in the history of life than the primeval, ΔpH-utilizing machines. The RML groups properly recognize therefore that primitive protein forms of the H^+^-ATP synthase and the H^+^-pyrophosphate synthase should be eliminated from consideration, as the early molecular machines driven by natural pH gradients (Fig. 1b). We may also rule out an ATP synthase on the basis that the reaction substrate and product are nucleotides—later arrivals in life’s origin in the RML scheme. However, it is simplistic to argue that non-protein/inorganic equivalents of these machines with unspecified structures, and utilizing completely unknown chemistry, could have served as consumers of those first putative natural pH gradients (Russell and Hall 2002; Russell 2007; Nitschke and Russell 2009; Russell et al. 2014). The mechanisms of the modern-day H^+^-pyrophosphate synthase (Lin et al. 2012) and H^+^-ATP synthase (Walker 1998) centrally require *extensive and complex alterations in protein conformation* to effect coupling between the hydration/dehydration chemistry and transmembrane proton translocation. These conformational changes, taking place across the protein molecule, are essential both to gate steps in the reaction and to support changes in substrate-binding energy. It is extremely difficult, if not impossible, to envisage how early, *non*-*protein*/inorganic versions of these enzymes could have operated without equivalent structural alterations. Speculations that “positive redox polaron waves….augmented by the proton gradient” in complexes of brucite, mackinawite- and fougèrite-like minerals can serve as a peristaltic drive of phosphate molecules into low-entropy two-dimensional space where they can condense to pyrophosphate (Russell et al. 2014) are splendidly imaginative, but not in the least supported by observations in the laboratory.

**Fig. 3:**
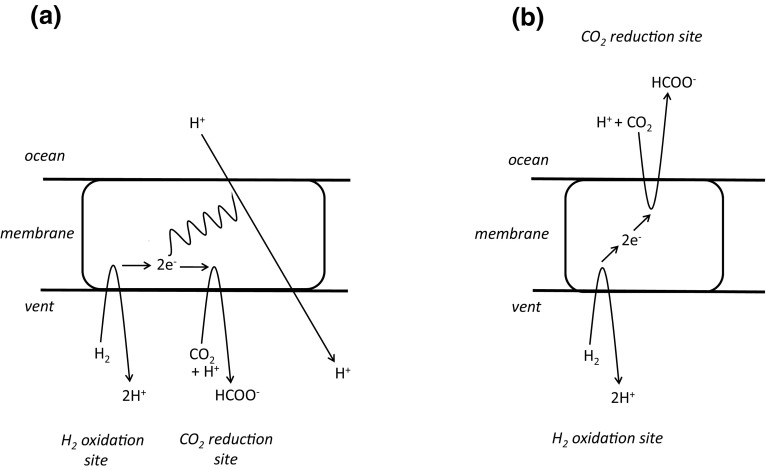
Models of a hypothetical machine catalysing H_2_-dependent reduction of CO_2_ to formate (HCF) in primeval vent membranes. See text. The *horizontal black lines* represent the inorganic membrane separating the vent fluid from the Hadean ocean. The thickness of the membrane is drawn conveniently to accommodate the diagram of the molecular machine. The question as to the relative size of the machine and the thickness of the membrane is addressed more cautiously in the text and in Fig. 4. The *wavy line* represents the conformational change linking the redox reaction to the proton translocation machinery. **a** Re-drawn from Nitschke and Russell (2009). **b** Re-drawn from Sojo et al. (2016)

In modern-day organisms, Δ*p* is used in some circumstances to drive “uphill” redox reactions, from an electron donor poised at relatively high Eh to an acceptor poised at relatively low Eh. A protein in which such a reaction takes place may be called a Δ*p*-consuming redox machine. Within the context of the RML hypothesis, a non-protein version of a Δ*p*- (or ΔpH-) driven redox machine might be a better proposition as a consumer of the primeval natural pH gradient than, for example, a pyrophosphate synthase because, in principle, protonation/deprotonation accompanying reduction/oxidation reactions in such a machine can be located on opposite sides of the membrane, and their operation can lead to a formal proton translocation without involving a large conformational change. The RML groups have often expressed interest in a redox machine driven by the natural ΔpH perhaps partly because it accords with their (not uncontentions) philosophy that a mineral-catalysed autotrophic metabolism preceded the RNA world and/or the RNA/protein world. A non-protein machine catalysing H_2_-dependent reduction of CO_2_ to formate (and here designated HCF) has, in fact, been suggested as a consumer of the natural ΔpH at the start of life (Russell 2007; Martin and Russell 2007; Nitschke and Russell 2009; Sojo et al. 2016). We shall accept, in principle, this possibility of HCF as a ΔpH-utilizing molecular machine, and briefly comment on mechanistic alternatives. In fact, two different mechanisms for a ΔpH-consuming HCF have been proposed (Nitschke and Russell 2009; Sojo et al. 2016), but only the second avoids the requirement for conformational changes that link the redox reaction with proton translocation (Fig. 3).

Nitschke and Russell (2009) suggested that the ancient, non-protein HCF might have parallels with the modern-day bacterial enzyme, formate hydrogenlyase (FHL), which catalyses the reverse reaction, the disproportionation of formate to CO_2_ and H_2_ accompanied by outward H^+^ translocation across the cytoplasmic membrane (Kim et al. 2010). The mechanism of action of this enzyme is barely understood, but evidence shows that both its formate oxidation site and its H^+^ reduction site lie on the *same side* of the membrane (the cytoplasmic side (McDowall et al. 2014), and proton translocation through the membrane-spanning components must therefore be coupled to the redox reaction by protein conformational changes—compare, for example, with the related and better understood mechanism of Complex I (Sazanov 2015). An equivalent mechanism in the primitive HCF (Nitschke and Russell 2009) with both the H_2_ oxidation and CO_2_ reduction sites on the same (vent) side of the membrane (Fig. 3a), and with conformationally-coupled H^+^ translocation is, as we argued above for an ATP synthase and pyrophosphate synthase, probably too much to expect in a population of non-protein molecular machines assembled by chance in the prebiotic environment: this is a black-box mechanism without the box.

The sister group (Sojo et al. 2016) offered what might be a more promising suggestion for the prebiotic HCF. Structurally, their model deviates substantially from what we know of the modern FHL, but perhaps we should steel ourselves not to be disturbed by this. The Sojo et al. (2016) model for the ΔpH-driven machine places the H_2_ oxidation site of HCF on the vent side of the membrane, and the CO_2_ reduction site on the ocean side, with intervening FeS providing an electron transfer pathway between them (Fig. 3b). The redox potentials of the H^+^/H_2_ and the CO_2_/formate couples are shifted by the alkaline vent fluid and the acidic sea water, respectively, such that H_2_ reduction of CO_2_ to formate becomes thermodynamically favourable. H^+^ release into the vent accompanying H_2_ oxidation and H^+^ uptake from the ocean accompanying CO_2_ reduction lead to a formal proton translocation. Large conformational changes are not required to effect H^+^ translocation in this model. In passing, we observe that the build-up of Δ*φ* (positive on the vent side) resulting from electrogenic electron transfer between the two sites would restrict the reaction and must be dissipated by electrophoretic movement of ions across the inorganic membrane (viz the ionic conductance path in Fig. 1b). An additional consideration is that (unlike in the Nitschke and Russell (2009) model, Fig. 3a) the production of formate takes place on the ocean side of the membrane (Fig. 3b) and, if not constrained, this product will diffuse away and be lost from the system—the subsequent reduction of formate to “CH_2_O” for “use” by the prebiotic system (Sojo et al. 2016) becomes difficult. Nevertheless, on mechanistic grounds, this is a much simpler non-protein molecular machine than that of Nitschke and Russell (2009), and mitigates the need for complex, conformational coupling between the redox reactions and proton translocation.

Sojo et al. (2016) and Nitschke and Russell (2009) both propose that the cores of their HCF models could have been based on a cluster of Fe, S, Ni and Mo atoms. A simple, crystalline array such as that found in the minerals greigite and mackinawite would not have sufficed for the machine—the cluster could not have been a wholly regular assembly since it had to encompass elementary structures for the substrate-binding sites and for binding the transition states (e.g. for the CO_2_/formate reaction). The chance assembly of such a machine, particularly in multiple copies, before emergence of gene encoded proteins would, of course, have been unlikely, astronomically so if the cluster were large. The RML groups point to well-documented structures of metal centres in modern-day redox proteins, which they say may be relics of the structures of molecular machines in the prebiotic era. Within their conceptual framework, we shall accept this as a guide to the size of the putative early molecular machine—perhaps it would have comprised a few tens or hundreds of atoms, and thus have been in the order of 1 nm in diameter, certainly smaller than the >10 nm of a modern-day membrane protein catalysing Δ*p*-linked redox chemistry.

## Focussing a Natural pH Gradient

Within the context of the RML hypothesis, it is important to emphasize that, to make full use of the available energy, the pH difference between the alkali vent fluid and the bulk water of the ocean must be focussed across the molecular machine—ultimately it is the pH gradient across the molecular machine rather than that across the membrane which is the critical parameter. To access the maximum ΔpH, the aqueous milieu on one side of the machine should have a pH close to that of the Hadean ocean, the other close to that of the vent. There is little energy to spare. In the context of the hypothesis, the function of the membrane may be thought of as helping to focus the natural pH gradient on the molecular machine.

### The Importance of the Relative Size of the Δ*p*-Driven Molecular Machine and the Thickness of the Membrane

**Fig. 4:**
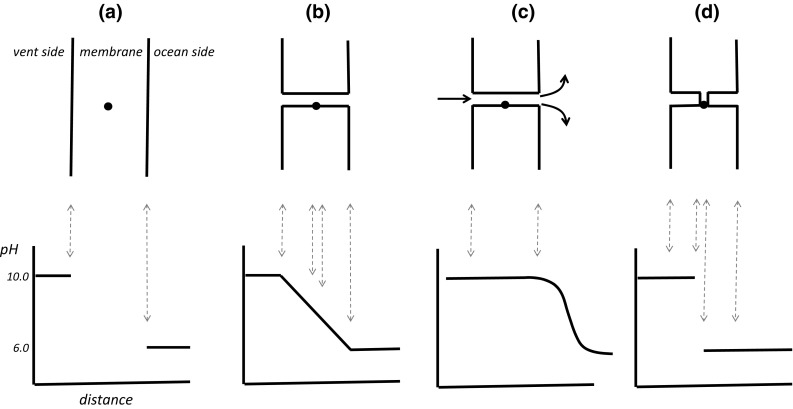
Profiles of pH along channels through a thick, inorganic membrane separating an alkaline vent from the ocean. The *top parts* of the figure show different diagrammatical sections through an approximately 1-μm-thick membrane. The molecular machine (•), of diameter approximately 1 nm (not to scale), is positioned near the centre of the membrane. The lower parts of the figure show illustrative pH profiles through the bulk aqueous phases and along channels in the membrane, referenced to the *top parts* by the *dashed vertical arrows.* The *heavy arrows* in (**c**) represent convective fluid flow through the channel and its mixing with the ocean water. **a** Non-porous and H^+^ impermeable. **b** Porous, H^+^ permeable, but no fluid flow. **c** Porous with fluid flow from vent to ocean. **d** Half channels

Figure 4a diagrammatically shows a small (e.g. 1 nm diameter) molecular machine located close to the centre of a (1 μm thick—see above) inorganic membrane. The machine is unable to access the ΔpH. The lower part of Fig. 4a shows a large pH gradient across the membrane (ΔpH = 4), but the pH within the membrane is undefined. Permeability channels would have been required to drive the machine. In the upper part of Fig. 4b, the molecular machine is located within a postulated channel which is permeable to H^+^ (and/or OH^−^). The pH drops in a monotonic function along the channel (lower part) due to diffusion of H^+^ from the ocean side to the vent side and of OH^−^ in the opposite direction. Although the machine can now access protons and hydroxide ions in the vent and the ocean, the ΔpH across the machine is much less than that between the bulk aqueous phases, only about 0.004 units in the simplified, example profiles of the lower part of Fig. 4b. From Eqs. 1 and 2, this amounts to an insignificant 24 J mol^−1^ protons transferred across the machine. For comparison, Fig. 4c shows the machine located within a channel or pore in which convective fluid flow from the vent to the ocean takes place at a rate somewhat faster than H^+^ and OH^−^ diffusion. In this situation, the pH along the channel remains close to that of the vent until the fluid mixes with the water of the ocean. An ill-defined pH gradient will then result on the ocean side dependent on the nature of the convective mixing. A molecular machine positioned anywhere along the channel of Fig. 4c is unable significantly to harness the energy of the bulk-to-bulk ΔpH.

Figure 4d shows that the RML hypothesis can be rescued from the dilemma of a reduced pH gradient if the molecular machine is located in a thin domain (e.g. approximately 1 nm thick) between two long (approximately 500 nm) proton-conducting half channels passing through the membrane, one linked to the vent and the other to the ocean. In this case, the pH remains constant along each half channel and drops across the locally thin region, and therefore across the molecular machine itself (lower part of the figure), and the energy of the full ΔpH can be harnessed. However, to achieve this organization, the molecular machine and the two half channels have to constructively engage. Either the machine has some special affinity with a hypothetical, thinned region of the membrane or the membrane undergoes spontaneous local thinning due to interaction with the machine. The improbability of a spontaneously-assembled inorganic machine is compounded with the improbability of channel construction. If the half channels described in Fig. 4d are connected to other channels in a labyrinthine fashion, then the pH profile along the channels more closely resembles that shown in Fig. 4b, and the system fails from an insufficient ΔpH across the machine. The requirements for a ΔpH-driven machine to operate in a thick membrane are exacting.

## General Discussion

The question we are addressing is whether it is likely that natural pH gradients had an obligatory role in the origin of life around 4 billion years ago. Observations at Lost City near the Mid-Atlantic Ridge show how serpentinization in the earth’s upper mantle can lead to alkalinisation of vent fluids emerging from the ocean floor, and we can tentatively accept the proposition that the ocean itself was slightly acidic in the Hadean era. Moreover, there are suggestions that such hydrothermal alkali fields were common below the oceans of the early earth (Kelley et al. 2002). However, these broad brush strokes draw us away from the more challenging molecular detail necessary to understand the origin of life.

Three issues have been discussed above which present severe problems for the natural pH gradient hypothesis. Firstly, there is the difficulty of interposing a suitable, stable, thin inorganic membrane between the alkaline vent fluid and the acidic ocean that could hold a sharp pH gradient. At Lost City, there is little or no evidence for the existence of stable inorganic membranes, let alone of membranes holding sharp pH gradients. Reports laying claim to the existence of thin mineral walls forming osmotic barriers between the alkali vents and the ocean at Lost City (Lane and Martin 2012) are an exaggeration of statements in the source publications (Kelley et al. 2001, 2005; Ludwig et al. 2006). Rather, the indications are that alkaline fluid passing through “stockwork” or “plumbing” systems in the underlying serpentinizing rock leaks or weeps from cracks and fissures, and thus mixes convectively and unpredictably with the ocean water forming friable precipitates of CaCO_3_ and Mg(OH)_2_. The possibility that gelatinous Fe(Ni)S membranes with appropriate properties (currently unknown and undefined) could have formed in a quite different vent environment (Russell and Hall 1997) stretches and weakens the Lost City analogy.

The second and even more difficult problem is that it is hard to accept that a molecular machine capable of abstracting useful energy from the natural ΔpH was assembled within the inorganic membrane by chance on the prebiotic earth, that is before the advent of gene encoded proteins and the great power of natural selection. Studies in structural biology during the last 50 years have revealed the subtlety and complexity of Δ*p*-utilizing protein machines. To assume that random chemistry could arrive at a primitive, functional, non-protein analogue on the prebiotic earth is probably unrealistic.

Thirdly, a simple, small, ΔpH-consuming molecular machine, even if by chance adopting an as yet unknown chemical structure, and a set of barely suspected, facilitating, mechanistic principles, will be unable to function in an inorganic membrane of a thickness deemed acceptable by the RML groups. To access the energy of a ΔpH, the molecular machine must equip itself, again by chance, with proton-conducting channels (of unknown, non-protein construction) linking with the two aqueous phases on either side of the relatively thick membrane.

The idea of a “free” pH gradient may be appealing but understanding how the energy of that gradient could have been harvested is another matter entirely. If this hypothesis is to retain credibility, experimental investigations can represent the only way forward. A direct demonstration that a model, laboratory-synthesized, non-protein, molecular machine can utilize a ΔpH established across a laboratory-synthesized, inorganic membrane is needed.

The RML groups have maintained that natural pH gradients had an important role at the very origin of life. They propose that the production of formate (or an equivalent molecule) by a ΔpH-utilizing molecular machine, was integrated with other reactions in an autotrophic protometabolism. Thus, a cartoon in Sojo et al. (2016) shows formate produced by HCF not escaping into the ocean, as expected, but being captured and further reduced to CH_2_O (a generic carbohydrate) by a *second* population of randomly assembled, ΔpH-utilizing molecular machines of different structure and function. The difficulties for the hypothesis are compounded. With the vents providing additional small molecules, local mineral surfaces are proposed to have served as catalysts for numerous other (though not necessarily ΔpH utilizing) reactions. Some of these would by chance have connected and given rise to the self-sustaining protometabolic network. Whether such a random assembly of reactions could have become organized and provided a pathway to the origin of life in itself raises substantial difficulties (see Orgel 2008).

There are alternative explanations for the origin of chemiosmotic energy transduction systems in living organisms, but these are set in the post-RNA world. Koch (1985) and Koch and Schmidt (1991) suggested that electron transport through metal centres embedded in the *lipid bilayer* membrane of a protocell, and linking oxidation/reduction reactions on either side of that membrane, could have served as the first *generator* of Δ*p*. The Δ*p* was used perhaps initially to drive weak acids and weak bases across the membrane (for use in metabolism), and subsequently to power newly evolved molecular machines such as the H^+^-ATP synthase. In the opinion of Koch and Schmidt (1991), the emergence of this chemiosmotic proton circuit took place some considerable time *after* the origin of life. Their concept of a Δ*p* generator is not unlike that of the non-protein Δ*p* consumer (HCF) later described by Sojo et al. (2016), but working in the opposite direction. However, significantly in the hypothesis of Koch and Schmidt (1991), this development would have taken place after the emergence of error-prone gene replication in primitive living cells, and would therefore have enabled selection, and thus refinement, of molecular structures and mechanisms. Working within a similar time frame, another possibility for the origin of chemiosmotic coupling arises from a perceived need to pump Na^+^ from early living cells (Mulkidjanian et al. 2008; Dibrova et al. 2012). It was suggested that the protein Na^+^ pump was the evolutionary forerunner of the H^+^-ATP synthase. Later, instead of pumping Na^+^, this protein began to operate in the reverse direction and thus to *use* Na^+^ electrochemical gradients to drive ATP synthesis. In some situations, a variant of the protein then changed its specificity following random genetic mutation and selection, and gained the capacity to use H^+^ electrochemical gradients generated by primitive, proton-translocating respiratory complexes.

Lane et al. (2010) disparage theories invoking the late arrival of chemiosmotic coupling in the protein/DNA world as “little more than an addendum incorporated at an arbitrarily late point”, and Martin (2012) describes such theories as a “casual afterthought”. Perhaps, a late arrival may be thought less radical or less heroic. Certainly, we might imagine very simple, non-ribozymal, non-protein, transition metal-based molecular machines, catalysing single redox steps, before the arrival of gene replication and translation. Thus, the electrochemical reduction of CO_2_ to CO, formic acid, acetic acid, pyruvic acid and methane on Fe(Ni)S mineral surfaces has been well documented (Yamaguchi et al. 2014; Roldan et al. 2015). However, to predict the appearance of even relatively primitive mechanisms for coupling such simple redox reactions to proton translocation in that very early period is probably beyond the pale. At a later stage of evolution, the assembly of *protein modules* into machines capable of new and more complex functions in lipid membranes became possible with the advent of genetic selection.
